# Birth outcomes in a Swedish population of women reporting a history of violence including domestic violence during pregnancy: a longitudinal cohort study

**DOI:** 10.1186/s12884-020-02864-5

**Published:** 2020-03-26

**Authors:** Hafrún Finnbogadóttir, Kathleen Baird, Li Thies-Lagergren

**Affiliations:** 1 Faculty of Health and Society, Department of Care Science, Malmö University, Malmö, Sweden; 2 School of Nursing and Midwifery, Transforming Maternity Care Collaborative, Griffith University, & Gold Coast University Hospital, Griffith, Australia; 3 The Department of Health Science: Midwifery research - reproductive, perinatal and sexual health, Health Science Center, Lund University, Lund, Sweden

**Keywords:** History of violence, Domestic violence, Intimate partner violence, Pregnancy, Midwife, birth outcomes, Longitudinal, Cohort-study

## Abstract

**Background:**

Victimisation of women is encountered in all countries across the world, it damages the mental and physical health of women. During pregnancy and the postpartum period, women are at a greater risk of experiencing violence from an intimate partner. The aim of this study was to explore childbirth outcomes in a Swedish population of women reporting a history of violence including domestic violence during pregnancy.

**Methods:**

A longitudinal cohort design was used. In total, 1939 pregnant women ≥18 years were recruited to answer two questionnaires, both questionnaires were administered in the early and late stages of their pregnancy. The available dataset included birth records of 1694 mothers who gave birth between June 2012 and April 2014. Statistical analyses included descriptive statistics, T-test and bivariate logistic regression.

**Results:**

Of 1694 mothers 38.7% (*n* = 656) reported a history of violence and 2% (*n* = 34) also experienced domestic violence during pregnancy. Women who were single, living apart from their partner, unemployed, smoked and faced financial distress were at a higher risk of experiencing violence (*p* = 0.001). They also had significant low scores on the SOC-scale and high EDS-scores ≥13 (*p* = 0.001) when compared to women without a history of violence (*p* = 0.001). Having a history of violence increased the woman’s risk of undergoing a caesarean section (OR 1.33, 95% CI 1.02–1.70). A history of emotional abuse also significantly increased the risk of having a caesarean section irrespective of whether it was a planned or an emergency caesarean section (OR 1.50, 95% CI 1.09–2.06). Infants born to a mother who reported a history of violence, were at significant risk of being born premature < 37 weeks of gestation compared to infants born by mothers with no history of violence (*p* = 0,049).

**Conclusions:**

A history of violence and/or exclusively a history of emotional abuse has a negative impact on childbirth outcomes including caesarean section and premature birth. Therefore, early identification of a history of or ongoing violence is crucial to provide women with extra support which may have positive impact on her birth outcomes.

## Background

Violence against women (VAW) continues to be one of the biggest violations against human rights with every third woman worldwide being exposed to physical and/or sexual violence [1]. Women living in Sweden are no exception to such statistics [2, 3]. Pregnancy is not a safeguard or protector against intimate partner (IPV) or family violence [4–6].

In this study, the terms ‘history of violence’, Domestic Violence (DV), Intimate Partner Violence (IPV) and the concept of ‘domestic and family violence’ (DFV), will be used interchangeable to describe DV. “A history of violence is defined as a lifetime experience of emotional, physical or sexual abuse occurring during childhood (< 18 years), adulthood (≥ 18 years) or both, regardless of the level of abuse or the perpetrator’s identity” [3]. The definition of DV used in the study is in agreement with the World Health Organization (WHO) description [7]. DV commonly takes place in the woman’s ´own home’ [8].

Home is often considered as a place of safety, a haven for women and children, a place where there is physical and psychological safety, trust and care [9]. However, for many women, what should be a place of safety, becomes an environment which is characterised by threat, fear and victimisation for many women experiencing DV [10]. Over the past two decades, research has highlighted the multidimensional effects of DV [11]. Women who experience DV have more medical and stress related symptoms, higher risk of substance misuse than non-abused women. It is also known that women living with violence are higher users of health care services with an estimated 30–50% higher use rate of Emergency Department [12]. Despite this, the response from healthcare in relation to DV is often inadequate and DV remains undetected also for pregnant women [13].

According to WHO, globally 1 to 28% of women are physically abused during pregnancy by an intimate partner [14]. In Australia, 36% of women who experienced violence by a partner reported that this occurred when they were pregnant and around 17 % of these women experienced, violence for the very first time during pregnancy [15]. A meta-analysis by James and colleagues exposed an overall prevalence rate of 13.3% of DV during pregnancy in high-income countries [16]. In Europe, 20% of pregnant women experienced IPV from a current partner with 42% of the women reporting IPV from a previous partner [17]. Whereas in Sweden, recent results from a longitudinal cohort study completed in southwest Sweden revealed a DV prevalence rate of 2.5% of the pregnant population [18, 19].

A growing body of evidence exploring the effects of IPV during pregnancy confirms that it leads to an increase in behavioural risk factors associated with adverse birth outcomes, such as maternal smoking, alcohol or drug misuse, inadequate prenatal care, or insufficient prenatal weight gain [20, 21]. Women who are subjected to IPV are predisposed to have higher levels of stress-related hormones which can be accelerated through physical and sexual trauma leading to preterm birth and low birth weight [22] resulting in reducing mother’s and child’s immune system [21]. A history of sexual violence may lead to fear of childbirth [23, 24] increasing the risk of both planned and emergency caesarean section (CS) [24].

In addition, being exposed to violence and abuse during pregnancy facilitates direct and indirect path-ways to adverse birth outcomes [20]. For example, placental damage, uterine contractions, premature rupture of membranes, and genitourinary infections are among some of the pregnancy complications and adverse outcomes caused by direct physical assault and sexual assault [21]. Women exposed to DV during pregnancy and the postpartum period are also at a greater risk of lifelong health consequences [22]. The condition of the mother’s health and wellbeing is also reflected in the health and wellbeing of the foetus and the new-born infant [25]. Consequences of abuse for maternal, foetal, and child health outcome is poorer physical and psychological health during pregnancy [26–28]. International literature has also revealed that women who are exposed to DV have a significantly increased risk for depression during both prenatal and postpartum period [28].

Currently, in Sweden, there continues to be limited knowledge relating to birth outcomes in women who have been subjected to DV before and/or during pregnancy. Consequently, the overall aim of this study is to explore childbirth outcomes in a Swedish population of women reporting a history of violence including domestic violence during pregnancy.

## Methods

The study used a longitudinal design and is part of a previous published project ‘*Pregnant women and new mothers’ health and life experience, with ‘life experience’ which* also included collecting data around intimate partner violence [3, 18, 19]. Included participants were primigravida and multiparous women ≥18 years of age, receiving antenatal care (ANC), and who could read and write in Swedish or English.

### Participants

Recruitment to the study was performed prospectively between March 2012 and September 2013 area in the southwest of Sweden, which is multiethnic. The study population includes all pregnant women at total 17 ANCs situated in the multiethnic city, a University City and in smaller municipalities. In the present study, more than 50% of the women lived in the multicultural city were almost a third of the inhabitants are born abroad and come from 186 different countries. Overall, the participants in the present study represented 93 different countries. Altogether, 1939 pregnant women were recruited to the study. Recruitment usually occurred early in the second trimester (mean, 12.8, SD 5.11) [3] by midwives working in maternal health care. Further details regarding the recruitment process and the study setting are described in detail in another paper [3].

### Data collection

**Fig. 1:**
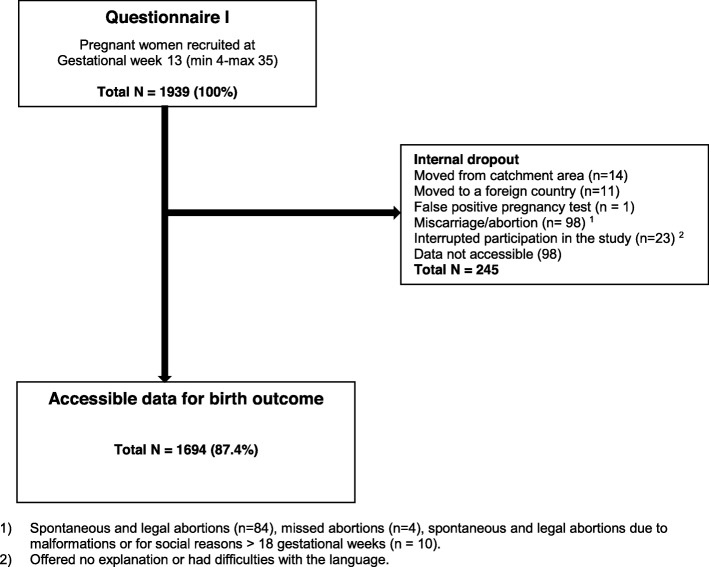
Flowchart over distributed and received answers from Questionnaires I-II and birth files

The available dataset included in the study involved the health records of 1694 mothers who gave birth between June 2012 and April 2014 (Fig. 1).

### Survey and tools

Questionnaires (QI and QII) were administered and completed early in the second trimester and again in late pregnancy, usually around gestational week 34 (mean 33.9; SD 2.20) information was also extracted from the birth register regarding the women’s birth outcomes.

Questionnaires included (Q-I – Q-II) the *NorVold Abuse Questionnaire* (NorAQ) which has been used previously for collecting similar data demonstrating its validity and reliability [29]. Other questionnaires included *Edinburgh Postnatal Depression Scale* (EPDS) [30], which is common tool used during pregnancy (EDS) and following childbirth to access a woman’s risk of developing postnatal depression [31]. The EPDS has a 72% sensitivity and 88% specificity for women in the postpartum period but has a lower degree of detection for depression during pregnancy [32]. The cut-off score for depression is usually set at 12/13 [30] the cut off chosen for this study was 13. *The sense of coherence* (SOC) scale measured the women’s’ stress management and their use of own resources to maintain and improve health. The instrument is reliable, valid and cross-culturally appropriate with acceptable face validity and consists of 13 items [33]*.* A high SOC score is a predictor of good health and is strongly related to perceived health, especially mental health [34]. *The Alcohol Use Disorders Identification Test* (AUDIT) [35] was also used at each time of questionnaire administration (Q-I – Q-II). For this study, the women were only asked the first question; *How often do you have a drink containing alcohol?* In Sweden, the AUDIT questionnaire is routinely used in antenatal care to collect information about the woman’s alcohol intake during pregnancy.

### Classification of the variables

*For sociodemographic factors as well as maternal* characteristics similar or same classifications were used as in previous publications, but the material has same origin [3, 18, 19]. *Age* was classified as 18–25, 26–34 and ≥ 35 years. *Cohabiting status* was classified as single/living apart, or common law spouse/married. *Language* was dichotomised as Swedish language or foreign language spoken at home (including English). *Educational status* was classified as compulsory school or less to *low educational status* and, high school or less, or university to *high educational status*. *Employment status* was dichotomised as employed (including parental leave and studying) or unemployed (including long illness). *Financial distress* was dichotomised as “no” (no problem) or “yes” (serious financial distress). The question about financial distress was as follows; *If you received unexpected bill of 20.000 SEK, how easy would it be for you to pay within a week?* Responses included; no problem, fairly hard and very hard, which is expressed as ‘serious financial distress’.

Maternal characteristics included *Parity* which was classified as primiparous versus multiparous. *Body mass index* (BMI) were calculated from maternal weight and height before the pregnancy and classified according to WHO’s definition as underweight (< 18.5) / normal weight (18.50–24.99) versus overweight (≥25–29.99) / obese (≥30). *Smoking/using wet tobacco* was dichotomised into “yes” (if the woman was a daily smoker/snuffer or smoking/snuffed at some point during pregnancy) and “no” (never smoked/wet-tobacco or ceased before pregnancy). *Use of alcohol* was dichotomised into “yes” (at least once a month or more) or “no”. *Fear of Birth* yes or no.

For birth and labour outcome *Labour initiation* was dichotomized into spontaneous or induction (regardless of diagnosis or how the induction was initiated). *Augmentation of labour* was dichotomized into yes (with synthetic oxytocin) or no. The use of *Epidural anaesthesia* was dichotomized into yes or no. *Birth mode* were classified as vaginal birth (inclusive vacuum extraction and forceps) or caesarean section (inclusive planned and emergency section). *Postpartum haemorrhage* (PPH) was classified as bleeding < 1000 ml or ≥ 1000 ml.

For infant characteristics and birth outcome *gestational week* was classified as premature < 37 and as full term from ≥37 weeks of gestation. Infant biological sex was dichotomized into female or male. *Apgar* scores at 5 min classified as < 7 or ≥ 7. Infant weight was distributed between < 2500 g, 2500-4000 g and >  4000 g. Transferred to Neonatal Intensive Care Unit (NICU), was dichotomized into yes or no.

### Data analysis

Descriptive statistics were utilized to show prevalence. The *t-*test was used to compute mean age. Chi-square analysis was used to investigate differences in variables presenting sociodemographic, maternal characteristics, birth and labor outcome as well as infant characteristics in relation to ‘history of violence’*.* OR and 95% CI were calculated for the crude associations between possible risk factors and ‘history of violence’, with ‘birth outcome’ as a dependent variable for bivariate logistic regression. In order to analyze the association between the SOC score and exposure to ‘history of violence’, the SOC-scale was dichotomized utilizing the first quartile of the distribution as a cut-off value (SOC ≤ 64 and SOC > 64) [36]. The SOC score was only subtracted for those responding to all thirteen items (missing = 95). To analyze the association between symptoms of depression during pregnancy an optimal cut-off of ≥13 was chosen as representing the presence of symptoms of depression [31]. The EDS score was calculated solely for those replying to all ten questions (missing = 53). Statistical significance was considered at *p* < 0.05 (two-tailed). Statistical analyses were performed using the Statistical Package for Social Sciences (SPSS) version 25.0 for Windows.

## Results

Accessible data to investigate for birth outcomes comprised of 87.4% (*n* = 1694) of the original cohort (*N* = 1939). The mean age (at recruitment) was 30 years (mean 30.12, SD 4.8; min 18–48 years) and for the majority of women Swedish was their first language. Among those women, almost 4 in 10 women reported a history of violence (*n* = 656), categories of significant difference were single or living apart, unemployed and experiencing financial distress (Table 1). In this particular cohort 2% (*n* = 34) women reported experiencing DV during pregnancy (Q-I – Q-II) and all had a history of experiencing violence.

**Table 1:** Socio-demographic factors at recruitment in early second trimester (*N* = 1694)

Characteristics	Total	History of violence ^1^		*P*
Total n (%)		No	Yes	
	n (%) 1687 (100)	n (%) 1031 (61.1)	n (%) 656 (38.9)	
Age, years				0.426
18–25	293 (17.6)	178 (17.4)	115 (17.9)	
26–34	1067 (64.1)	666 (65.2)	401 (62.4)	
≥ 35	305 (18.3)	178 (17.4)	127 (19.8)	
*Missing = 29*				
Cohabiting status				0.001
Single/Living apart	84 (5.1)	30 (3.0)	54 (8.5)	
Common law spouse/married	1554 (94.9)	969 (97.0)	585 (91.5)	
*Missing = 56*				
Language				0.229
Swedish	1271 (75.5)	766 (74.5)	505 (77.1)	
Foreign language	412 (24.5)	262 (25.5)	150 (22.9)	
*Missing = 11*				
Educational status				0.107
Low educational status	555 (32.9)	324 (31.4)	231 (35.2)	
High educational status	1132 (67.1)	707 (68.6)	425 (64.8)	
*Missing = 7*				
Employment status				0.001
Employed Unemployed	1595 (94.6) 91 (5.4)	994 (96.5) 36 (3.5)	601 (91.6) 55 (8.4)	
*Missing = 8*				
Financial distress				0.001
No	886 (52.6)	584 (56.7)	302 (41.6)	
Yes	799 (47.4)	446 (43.3)	353 (53.9)	
*Missing = 9*				

Pre-specified level of statistical significance < 0.05, two-tailed

^1^Reported experience of emotional, physical or sexual abuse during pregnancy irrespective severity

In the self-reported maternal characteristics and lifestyle questionnaire, more women were multipara, of normal weight or were underweight with less than 4% reporting fear of birth. Smoking or used wet-tobacco were significantly higher among women with a history of violence. Exposed women also reported a significant lower SOC score and higher EDS scores than women who did not report a history of violence (OR 2.40, 95% CI (1.90–3.01) respectively (OR 2.41, 95% CI 1.71–3.40). Alcohol consumption were similar in the two groups, regardless of whether they reported a history of violence or not (Table 2).

**Table 2:** Descriptive overview of maternal characteristics, in early second trimester of the pregnancy (*N* = 1694)

Characteristics	Total	History of violence^1^		*P*
Total n (%)		No	Yes	
	n (%)	n (%)	n (%)	
	1687 (100)	1031 (61.1)	656 (38.9)	
Parity				0.283
Primiparae	708 (45.2)	419 (44.2)	289 (46.9)	
Multiparae	857 (54.8)	530 (55.8)	327 (53.1)	
*Missing = 129*				
BMI				0.411
Underweight/ Normal weight	1199 (73.8)	742 (74.5)	457 (72.7)	
Overweight/ Obese	426 (26.2)	254 (25.5)	172 (27.3)	
*Missing = 69*				
Smoking/Wet-tobacco				0.001
No	1338 (81.6)	860 (85.9)	478 (74.8)	
Yes	302 (18.4)	141 (14.1)	161 (25.2)	
*Missing = 54*				
Use of Alcohol				0.542
No	773 (47.4)	467 (46.8)	306 (48.3)	
Yes^2^	858 (52.6)	531 (53.2)	327 (51.7)	
*Missing = 63*				
Fear of birth				0.159
No	1625 (96.4)	998 (96.9)	627 (95.6)	
Yes	61 (3.6)	32 (3.1)	29 (4.4)	
*Missing = 8*				
SOC				0.001
High score	1194 (74.7)	782 (81.4)	412 (64.6)	
Low score	405 (25.3)	179 (18.6)	226 (35.4)	
*Missing = 95*				
EDS				0.001
Score < 13	1492 (90.9)	934 (93.9)	558 (86.4)	
Score ≥ 13	149 (9.1)	61 (6.1)	88 (13.6)	
*Missing = 53*				

Pre-specified level of statistical significance < 0.05, two-tailed

^1^Reported experience of emotional, physical or sexual abuse during pregnancy irrespective severity

^2^At least once in a month

Maternal birth outcomes were similar in both groups apart from CS, women who had a history of violence had a 33% increased risk of CS (OR 1.33, 95% CI 1.02–1.70) (Table 3). In the total 15.3% (*n* = 259) of the study population experienced a CS. They were divided between 5.8% (*n* = 99) women who chose a planned CS and 9.4% (*n* = 160) who had an emergency CS (exclusively presented in the text). Women with history of emotional abuse had significantly increased risk having CS (OR 1.50, 95% CI 1.09–2.06) (Table 4). There was also an increased risk of prematurity for women who were experiencing history of violence 6.6% (*n* = 43) compared to those who were not 4.4. % (*n* = 45) (Table 5).

**Table 3:** Maternal labour and birth outcome (*N* = 1694)

Characteristics	Total	History of violence^1^		*P*
Total n (%)		No	Yes	
	n (%) 1687 (100)	n (%) 1031 (61.1)	n (%) 656 (38.9)	
Labour initiation				0.136
Spontaneous	1384 (85.1)	856 (86.1)	528 (83.4)	
Induction	243 (14.9)	138 (13.9)	105 (16.6)	
*Missing = 67*				
Augmentation				0.946
No	1058 (68.5)	649 (68.5)	409 (68.6)	
Yes	486 (31.5)	299 (31.5)	187 (31.4)	
*Missing = 150*				
Epidural anesthesia				0.067
No	1267 (78.5)	788 (80.0)	479 (76.2)	
Yes	347 (21.5)	197 (20.0)	150 (23.8)	
*Missing = 80*				
Labour outcome				0.036
Vaginal birth^**2**^	1430 (84.8)	889 (86.2)	541 (82.5)	
Caesarean section	257 (15.2)	142 (13.8)	115 (17.5)	
*Missing = 7*				
Post partum hemorrhage				0.259
< 1000 ml	1602 (95.0)	984 (95.4)	618 (94.2)	
≥ 1000 ml	85 (5.0)	47 (4.6)	38 (5.8)	
*Missing = 7*				

Pre-specified level of statistical significance < 0.05, two-tailed

^1^Has reported experience of emotional, physical or sexual abuse during pregnancy irrespective severity

^2^Inclusive vacuum extraction (*n* = 121) and forceps (*n* = 10)

**Table 4:** Caesarean section in relation to history of violence (*N* = 1694)

Characteristics	Total	Caesarean sectio		*P*
Total n (%)		No	Yes	
	N (%)	n (%)	n (%)	
	1694 (100)	1435 (84.7)	259 (15.3)	
History of violence^**1**^				0.036
No	1031 (61.1)	889 (62.2)	142 (55.3)	
Yes	656 (38.9)	541 (37.8)	115 (44.7)	
*Missing = 5*				
DV during pregnancy^**2**^				0.924
No	1660 (98.0)	1406 (98.1)	254 (98.1)	
Yes	34 (2.0)	29 (2.0)	5 (1.9)	
*Missing* = 0				
History of emotional abuse^**3**^				0.012
No	1366 (81.4)	1173 (82.4)	193 (75.7)	
Yes	313 (18.6)	251 (17.6)	62 (24.3)	
*Missing = 15*				
History of physical abuse^**3**^				0.058
No	1193 (71.2)	1024 (72.1)	169 (66.3)	
Yes	482 (28.8)	396 (27.9)	86 (33.7)	
*Missing = 19*				
History of sexual abuse^**3**^				0.079
No	1424 (84.8)	1217 (85.5)	207 (81.2)	
Yes	255 (15.2)	207 (14.5)	48 (18.8)	
*Missing = 15*				

Pre-specified level of statistical significance < 0.05, two-tailed

^1^Reported experience of of emotional, physical or sexual abuse in early and/or late pregnancy irrespective severity

^2^Reported experience of of emotional, physical or sexual abuse during pregnancy irrespective severity

^3^Reported experience of emotional, physical or sexual abuse during early second trimester irrespective severity

**Table 5:** Infant characteristics and birth outcome (*N* = 1694)

Characteristics	Total	History of violence^1^		*P*
Total n (%)		No	Yes	
	n (%) 1687 (100)	n (%) 1031 (61.1)	n (%) 656 (38.9)	
Gestational week				0.049
Premature < 37 weeks	88 (5.2)	45 (4.4)	43 (6.6)	
Full term ≥37 weeks	1599 (94.8)	986 (95.6)	613 (93.4)	
*Missing = 7*				
Infant biological sex				0.212
Female	829 (49.3)	494 (48.1)	335 (51.2)	
Male	851 (50.7)	533 (51.9)	319 (48.8)	
*Missing = 13*				
Apgar at 5 min				0.634
< 7 ≥ 7	18 (1.1) 1669 (98.9)	10 (1.0) 1021 (99.0)	8 (1.2) 648 (98.8)	
*Missing = 7*				
Infant weight				0.742
< 2500 g	50 (3.0)	28 (2.7)	22 (3.4)	
2500–4000	1293 (77.3)	790 (77.4)	503 (77.3)	
> 4000 g	329 (19.7)	203 (19.9)	126 (19.4)	
*Missing = 22*				
Transferred to NICU				0.210
Yes	89 (5.9)	49 (5.3)	40 (6.8)	
No	1424 (94.1)	879 (94.7)	545 (93.2)	
*Missing = 181*				

Pre-specified level of statistical significance < 0.05, two-tailed

^1)^ Reported experience of emotional, physical or sexual abuse during pregnancy irrespective severity

Confounding effects of the increased incidence of lower socioeconomic status (cohabiting, employment, economy) and tobacco use on the rate of the two adverse pregnancy outcomes i.e. premature birth and CS showed no potential cause and effect or significant association.

Infant characteristics were similar in the two groups. In addition, there were two intra uterine foetal deaths before gestational week 34 (mothers had not reported experience of violence).

## Discussion

The findings from this study clearly indicate that women who are experiencing DV are at a significantly higher risk of a CS, irrespectively whether the CS is planned or emergency. In line with previous studies, women with a history of violence were also at an increased risk of a premature birth before 37 week of gestation. Tomasdóttir et al. [37] also reported that women with a history of violence were at an increased risk of a CS, however Tomasdóttir did not report on whether the CS was an emergency or planned. BIDENS large cohort study which was conducted in six European countries demonstrated the risk of a planned CS for non-obstetrical reasons in primiparous women who had experienced sexual abuse as an adult was high when compared with women who had not experienced sexual violence [24].

The same study also highlighted an association of an increased risk of preterm birth and DV, validating a recent prospective cohort study by Rishal et al. [38] and a large meta-analysis in 2016 by Hill et al. [21] which also demonstrated a significant increase risk of a preterm birth among women subjected to DV. The consequences of a preterm birth have short- and long-term consequences in infants. Infants born before 37 gestational weeks are vulnerable to major health and developmental risks such as increased risk of respiratory distress and immature brains requiring special care in NICU which in turn result in longer hospitalization [39]. Preterm infants are at higher risk of being readmitted to the hospital and are at an increased risk of death after returning home [40, 41]. In this study women who reported a history of violence experienced higher levels of depressive symptoms than women who were not experiencing DV. It is established that depression will influence a mother’s ability and willingness to bond and take care of her new-born child [42]. A systematic review by Staneva and colleagues reported a significant interface between depression, anxiety and stress, risk factors and preterm birth [43]. Previous research has also revealed that maternal psychosocial stress, and/or fear of childbirth, may have an association with specific complications and increase the women’s risk of undergoing a CS [44]. Pregnancy and childbirth can also be psychological memory triggers for women who have experienced childhood sexual abuse [45]. Simkin [46] supports this theory suggesting such complex psychosocial factors, whether remembered or not, have the capacity to play a greater role in perinatal care and outcomes. Finnbogadóttir and Mellgren [47] in a previous study from the same cohort of women revealed that more than one in ten women with a history of emotional abuse had never actually talked to anyone about their experience. Indicating that many women are embarking upon their pregnancy with unprocessed trauma which can adversely affect the woman’s pregnancy and childbirth outcome. However, in this study women with an exclusive history of physical and/or sexual abuse, did not display a significant association with increased risk of CS. This finding however, is in contraindication with some former studies which have shown an increased risk between IPV and CS and physical violence [48, 49]. Furthermore, a European a large multi-country cohort study in 2016 confirmed that primiparous women who were sexually abused as adults were more likely to have an elective CS for non-obstetrical reasons and multiparous for an emergency CS [24]. To summarize, irrespective of the type of violence a woman experiences a history of violence and ongoing abuse during pregnancy has an overall negative impact on childbirth outcomes. Therefore, it’s crucial for midwives to be focussed and observant during the antenatal visits with a pregnant woman. Therefore, it’s central that midwives are concentrating and observant in the antenatal period. It is also important that the midwife strives to develop a trusting relationship from the very first encounter and focuses on a woman’s holistic health needs. Thereby, providing an opportunity to identify the women who require extra resources and need extra support during such a vulnerable period of their lives.

Previous studies from Sweden testify that on average 2.6% of women have reported physical abuse by a current or former partner during the year preceding pregnancy, with 1.3% reporting a history of abuse in the postpartum period [50]. Evidence has shown that a brief training program can improve midwives’ ability to recognize the signs of DV, increase their knowledge, preparedness, and confidence to responding sensitively to women [51]. However currently in Sweden it is not routine to ask about DV. For that reason, this study sought to bring forward new knowledge to help midwives and other health care professionals recognize women who are a higher risk of experiencing DV during pregnancy. Pregnancy is an opportune time for early intervention, it is time when women may be open to accessing support. A disclosure which is met with a sensitive, caring and considered response may support and empower women to reach and seek support. Therefore, it is important that a woman-centred approach is secured, this can be achieved by providing care within a midwifery continuity of care model [52]. Providing midwifery continuity of care provides women with care from a known midwife. Continuity of midwifery care provides a greater level of trust and connection between a midwife and the woman thereby increasing the possibility of a disclosure from the woman. However, to provide women centred care, midwives must be supported by their health care organizations to re-organise their current fragmented systems of care. DV education and training is also vital to increase midwives’ knowledge and confidence, ongoing training, support and mentoring are vital if midwives are to continue to undertake this sensitive work in a trauma informed way. In addition, DV training programs should be long enough to address the complexities of DV, including addressing attitudes and stereotypes as well as ousting common myths, which surround DV. Continuity of midwifery care through midwifery caseload models is essential, as it provides women with the opportunity to build up a trusting and ongoing relationship with a known midwife. Continuity of midwifery care also offers a greater level of trust and connection between a midwife and the woman and increases the possibility of a disclosure from the woman. However, to provide women centred care, midwives must be supported by their health care organizations to re-organise their current systems of care. Education and training is also vital to increase the midwives’ knowledge and confidence, ongoing training, support and mentoring are vital if midwives are to continue to undertake this sensitive work. In addition, DV training program should be long enough to address the complexities of DV, including addressing attitudes and stereotypes as well as ousting common myths, which surround DV [51].

There are several strengths to this study the large sample size and the use of a well-defined cohort. This longitudinal study, based on prospective data, also allowed for the robust comparison between those who had history of violence and those who did not. Another strength of the study is the use of validated instruments to evaluate the data [29, 30, 33, 35, 53–55].

The authors would also like to acknowledge the limitations to the study, for example, the question *How often do you have a drink containing alcohol?* should have specified, when pregnant. The results are also limited to those who meet the inclusion criteria and were recruited to participate in the study.

## Conclusion

The results of the study ascertain that a history of violence has a negative impact on a woman’s mode of birth and increases the risk of premature birth. Therefore, it is important that midwives strive to develop a relationship which is respectful and built on trust with women during the antenatal period. Early identification of a history of violence and abuse before or during pregnancy could decrease the rates of premature birth and/or CS. The effects of a history of violence including experiencing psychological violence without other forms of violence has serious effects on both maternal and childbirth outcome.

## Data Availability

The datasets used and/or analyzed during the current study are available from the corresponding author on reasonable request.

## Abbreviations

ANC: Antenatal Care
BMI: Body Mass Index
CS: Caesarean Section
DV: Domestic Violence
EDS: Edinburgh Depression Scale
EPDS: Edinburgh Postnatal Depression Scale
IPV: Intimate Partner Violence
NorAQ: NorVold Abuse Questionnaire
Q-I: Questionnaire I
Q-II: Questionnaire II
OR: Odds ratios
SOC-13: Sense of Coherence Scale-short form
VAW: Violence Against Women
WHO: World Health Organization
